# Wearable Electromyography Classification of Epileptic Seizures: A Feasibility Study

**DOI:** 10.3390/bioengineering10060703

**Published:** 2023-06-09

**Authors:** Achraf Djemal, Dhouha Bouchaala, Ahmed Fakhfakh, Olfa Kanoun

**Affiliations:** 1 Measurement and Sensor Technology, Chemnitz University of Technology, Reichenhainer Straße 70, 09126 Chemnitz, Germany; 2 Laboratory of Signals, Systems, Artificial Intelligence and Networks, Digital Research Centre of Sfax, National School of Electronics and Telecommunications of Sfax, Technopole of Sfax, Ons City 3021, Tunisia; 3 National Engineering School of Sfax, University of Sfax, Route de la Soukra km 4, Sfax 3038, Tunisia

**Keywords:** epilepsy diagnosis, seizures classification, machine learning, features extraction, features selection, surface electromyography (sEMG), Big-O notation, wearable systems

## Abstract

Accurate diagnosis and classification of epileptic seizures can greatly support patient treatments. As many epileptic seizures are convulsive and have a motor component, the analysis of muscle activity can provide valuable information for seizure classification. Therefore, this paper present a feasibility study conducted on healthy volunteers, focusing on tracking epileptic seizures movements using surface electromyography signals (sEMG) measured on human limb muscles. For the experimental studies, first, compact wireless sensor nodes were developed for real-time measurement of sEMG on the gastrocnemius, flexor carpi ulnaris, biceps brachii, and quadriceps muscles on the right side and the left side. For the classification of the seizure, a machine learning model has been elaborated. The 16 common sEMG time-domain features were first extracted and examined with respect to discrimination and redundancy. This allowed the features to be classified into irrelevant features, important features, and redundant features. Redundant features were examined with the Big-O notation method and with the average execution time method to select the feature that leads to lower complexity and reduced processing time. The finally selected six features were explored using different machine learning classifiers to compare the resulting classification accuracy. The results show that the artificial neural network (ANN) model with the six features: IEMG, WAMP, MYOP, SE, SKEW, and WL, had the highest classification accuracy (99.95%). A further study confirms that all the chosen eight sensors are necessary to reach this high classification accuracy.

## 1. Introduction

One of the most prevalent neurological disorders is epilepsy, which affects about 50 new persons per 100,000 annually [1]. Unexpected and unprovoked seizures are a symptom of this complex neurological condition brought on by abnormally high or synchronized neuronal activity in the brain. A seizure results from the brain’s nerve cells firing out of control, and it might cause a convulsion, minor physical symptoms, mental confusion, or a mix of symptoms. A psychogenic non-epileptic seizure (PNES), which resembles an epileptic seizure but lacks the distinctive electrical discharges associated with epilepsy, is one example of a non-epileptic event that can be distinguished from an epileptic event using an epileptic seizure. Persons of all ages are impacted by the chronic, non-communicable brain disorder known as epilepsy. According to the World Health Organization (WHO), over 50 million persons worldwide (or about 1% of the population) suffer from epilepsy, with the majority of them living in developing countries [2]. According to the latest WHO data published in 2020, 0.43% of total deaths were caused by epilepsy in Lesotho. Mortality among people with epilepsy is up to three times higher than for the general population [1]. In addition, the annual rates of epilepsy misdiagnosis are still stubbornly high, which range from 2% to 71% [3]. For example, in the case of therapeutic epilepsy diagnosis in clinical practice, as well as drug trials, the seizure diagnosis is based on a self-reporting approach. This remains largely unreliable, where 47–63% of seizures are unrecognized by patients, and this is even higher (86%) for nocturnal seizures [4].

The treatment gap may be one of the reasons contributing to the higher mortality rate [1]. It is estimated by the WHO that up to 70% of persons living with epilepsy could live seizure-free if diagnosed and treated appropriately. For epilepsy diagnosis, a few marketed devices for epilepsy monitoring using surface EMG sensors are presented. The most common ones are non-invasive, wearable, and used as seizure detection systems. In [5], Conradsen et al. suggest a method for detecting generalized tonic-clonic (GTC) seizures. This algorithm has been modified and implemented in a small sEMG wireless device developed by DELTA, Denmark, on behalf of IctalCare A/S, Denmark. The wireless device for sEMG measurements was attached to the tibia muscle. The final results show a mean detection rate of 57%. According to reports, the device only sets hidden alarms; thus, medical workers are not aware of the times of the alarms. In [6], Bruno et al. highlight the SPEAC device used for adjunct seizure monitoring for adults. The device is positioned on the belly of the biceps muscle to analyze sEMG signals that may be associated with GTC seizures. The authors report that during the study, it became clear that the device had been improperly placed and was not correctly attached to the belly of the biceps, resulting in a detection rate of 76%. Whitmire et al. [7] employed the same system to identify GTCSs, and it was noted that the false-alarm rate ranged from 0.3 to 0.5 each day. There was no information on the seizure detection rate in this group compared to seizure diaries. SeizureLink, another surface EMG-based seizure detector utilized by Beniczky et al. [8], was formerly known as the Epileptic seizure Detector Developed by IctalCare (EDDI). The system needs to be fastened to the patient’s biceps and wirelessly connects to other devices to provide real-time alarms in the case of convulsive seizures and reach a detection rate of 93.8%. Most existing systems achieve acceptable performance in terms of sensitivity for detecting GTC seizures based on sEMG signals [9].

Detecting seizures is important for patients and their caregivers because it provides an opportunity for intervention. But, a gap for current devices is that they are tasked with detecting only generalized tonic-clonic seizures (binary classification). Although, it is essential to determine the type of seizure to guarantee an appropriate sufficient diagnosis and therapy.

Identifying the type of seizure, although sometimes difficult, is done through clinical observation with reference to patients’ medical history and demographic information. It is supported by general brain imaging techniques, such as electroencephalograph (EEG), magnetoencephalography (MEG), and functional magnetic resonance imaging (fMRI) [10,11]. Apart from these clinical observations, there is currently no portable device to assist in the classification of seizure types. Devices for seizure classification offer more precise seizure quantification, enabling doctors to customize treatment more objectively. Aside from this, however, electroencephalography (EEG) coupled with video surveillance is considered the most reliable and recognized analytical technique for diagnosing and classifying epilepsy [12,13]. Accurate seizure classification is important for patients, families, researchers, and medical professionals who care for persons with epilepsy and influences medication selection [14,15]. The accurate identification of the seizure type is challenging because of numerous factors. First, the clinical and EEG signs of different seizure types are similar. Even for a highly experienced neurologist, it can often be difficult to distinguish between focal and generalized seizures [3,16]. Second, in some cases, long-term monitoring (sometimes referred to as video-EEG monitoring) is required, and it may last for days to analyze these enormous records manually. Additionally, signal interpretation has a notoriously low inter-rater agreement, which is entirely dependent on the expert’s level of experience. Further, the inter-subject variability results in a variety of symptoms of the same type of seizures [17]. Moreover, patients must wear scalp electrodes and remain attached to EEG equipment during monitoring, which increases artifacts, is impractical, and potentially leads to stigmatization and discomfort [1,18]. In recent clinical studies, researchers have explored different methods for monitoring epileptic seizures. In level 3 [19], and level 4 [20] clinical studies recordings of EEG, 3D-accelerations and angular velocity have been used for monitoring epileptic seizures. Combining these different types of data can provide a more comprehensive view of the seizure and potentially improve diagnostic accuracy and treatment options. In this paper we pursue the same research path towards monitoring epileptic seizures and propose a new method based on wearable wireless EMG sensors monitoring the muscle activity and seizure classification method based on machine learning techniques. The suggested method involves multiple measurements of surface EMG data to categorize epileptic seizures according to typical seizure movements. This study highlights the technical nature of the research and emphasizes its preliminary nature as a feasibility study on healthy volunteers before progressing to clinical trials.

The paper is organized mainly into six sections. Section 2 presents an overview of epileptic seizure types and sensors used for an epilepsy diagnosis. Section 3 is devoted to the implementation of the wireless sensor node, data collection, and description. Section 4 highlights the processing steps to classify seizures, including feature extraction, selection step, and evaluation. Section 5 introduces different machine learning algorithms for classifying seizures and provides an evaluation of their performance. Finally, the conclusions of the work are presented in Section 6.

## 2. Epileptic Seizure Types and Sensors Used for the Diagnosis

**Figure 1:**
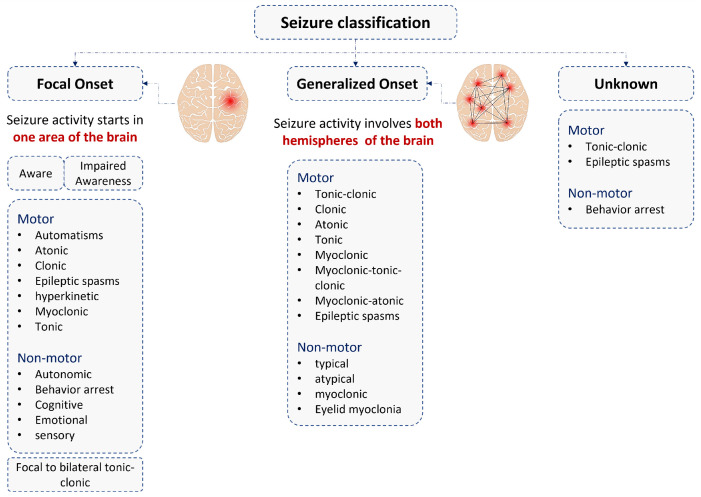
Epileptic seizure types.

Two main groups of epileptic seizures, which are focal and generalized, are presented in Figure 1 according to the International League Against Epilepsy (ILAE) categorization strategy for epileptic seizure classifications [21]. When abnormal electrical activity begins in one area of the brain, it is called a focal or partial seizure; however, generalized seizures begin on both sides of the brain [21]. The terms “motor” and “non-motor” are also used when describing seizure types. According to the Epilepsy Foundation, motor relates to physical movement or motion, and seizures involving motor activity may either increase or decrease muscle tone, leading to muscle twitches, jerks, or contractions. Non-motor onset seizures don’t involve muscle action but may include behavioral, emotional, and/or sensory activity or actions.

### 2.1. Epileptic Myoclonic Seizure

Myoclonic seizures are characterized by sudden, brief muscle contractions or twitches. The term “myoclonic” comes from the word “myo,” meaning muscle, and “clonus,” meaning rapidly alternating contraction and relaxation [22]. These seizures typically last for less than a couple of seconds [9]. They may occur singularly or in clusters. Some forms of epilepsy are referred to as syndromes due to their distinct signs and symptoms [23]. The type of seizures, age of onset, gender, behavior, and results from medical investigations and genetic testing may all be considered by doctors, as noted by the epilepsy foundation. Myoclonic seizures are more observed in the case of children, but can also occur in the case of adults as well. In fact, some individuals may continue to experience these types of seizures into adulthood, especially if they have an underlying neurological condition that predisposes them to this type of seizures [24]. Understanding whether a person’s epilepsy is linked to a syndrome can help in determining if their seizures can be controlled and in selecting the most appropriate diagnostic approach, either physiological or non-physiological [21].

Epilepsy patients can experience myoclonic seizures that result in coordinated, unusual movements across both sides of their body. These seizures can appear in a variety of epilepsy syndromes, each with its own unique characteristics, such as juvenile myoclonic, Lennox-Gastaut, and progressive myoclonic. The seizures associated with juvenile myoclonic syndrome typically affect the neck, shoulders, and upper arms and often occur shortly after waking up. Lennox-Gastaut syndrome is characterized by seizures that can be severe and difficult to control and affect the neck, shoulders, upper arms, and sometimes the face. Unfortunately, treatment for progressive myoclonic syndrome is typically ineffective as the condition tends to worsen over time and is not often seen.

### 2.2. Epileptic Tonic Seizure

Generalized tonic seizures are defined by the simultaneous tonic extension of both upper and lower limbs, giving the appearance of “decerebrate” posturing, as well as the simultaneous tonic flexion of the upper limbs and extension of the lower limbs, resembling “decorticate” posturing. They may also be accompanied by tremors in the extremities, according to [1]. The classification assumes that tonic activity is not followed by clonic movements. Tonic seizures are brief episodes, typically lasting less than 60 s, during which there is a sudden increase in muscle tone in the extensor muscles. They are generally of longer duration than myoclonic seizures and may also occur in the case of adults, particularly if they have a neurological condition that makes them more susceptible to these seizures [24].

Tonic seizures are commonly seen in patients with Lennox Gastaut syndrome and have been classified into four types: axial, axorhizomelic, global, and asymmetric. Axial tonic seizures are marked by a tightening of the neck muscles that causes the head to be held upright, the eyes to be wide open, and the jaw to clench or the mouth to open. This type of seizure is also accompanied by contraction of the respiratory and abdominal muscles, which may result in a high-pitched cry and brief pauses in breathing. Axorhizomelic seizures resemble axial tonic seizures, but the tonic contractions extend to the proximal muscles of the upper limbs, causing the shoulders to raise and the arms to be abducted. Global seizures are characterized by tonic contractions that affect the peripheral muscles of the limbs, causing the arms to be raised and clenched in front of the head, creating a defensive posture. Asymmetric tonic seizures can range from a slight head rotation to a tonic contraction of all the muscles on one side of the body.

### 2.3. Surface Electromyography (sEMG) and Quantity Analysis

Muscle movement is made under the control of our brain [25]. Thus, the electrical activity of muscles is very closely related to the nervous system. The brain produces an action potential, which passes through the nerve fibers. This action potential that passes through the nerve fibers will stimulate the muscle fibers. Motor neurons transmit electrical signals that cause muscles to contract. This causes the movement of the muscles. The electric potential from the muscles, which is represented in the form of a time-varying signal, is known to be the electromyography (EMG) signal [26]. Surface EMG (sEMG) is among the most promising physiological signals in the health monitoring field due to its flexibility, non-invasive method, large recording region, and high-quality measurement, which are essential properties for numerous clinical applications such as epilepsy diagnosis [27]. It is well demonstrated that the amplitude of the EMG signal is random and can be reasonably represented by a Gaussian distribution function. EMG’s amplitude is quite small. When the muscle does not contract, the amplitude of the EMG signal is generally in the range of [80 mV–90 mV]. However, the muscle contraction amplitude is only a few hundred mV at most [28]. So, in order to acquire an observable signal, the EMG signal is often amplified by 50–100 times to reach above 1–2 volt [26].

Muscles are the endpoints of the common final neural pathways involved in motor seizures. Thus, surface EMG signals provide valuable information on the Central Nervous System (CNS) activity during epileptic seizures [29]. Up to now, no data on quantitative EMG features during tonic or myoclonic seizures has been published. We hypothesized that quantitative EMG features would distinguish between the tonic and myoclonic phases. In addition, we also wanted to compare these phases to the normal state (no seizure) when no movement is simulated (EMG recording of the normal muscle activation in the rest position of the subject). Assessment of the EMG signals showed that the quantitative analysis of muscle activation differs from epileptic seizures and convulsive Psychogenic Non-Epileptic Seizures (PNES), even when both types of episodes occur in the same subject [29]. For that, the subject’s movements can be distinguished during both episodes. The tonic phase was characterized by a marked increase in amplitude-derived parameters; tonic seizure had a marked increase in frequency compared to myoclonic seizure for all muscles and was more straightforward for the lower limb muscles. Moreover, the coherence between the homologous muscles on the left and right sides was higher than during voluntary muscle activation [30,31,32,33,34]. Based on the quantitative analysis of the EMG signal, surface EMG proved to be an efficient tool for the classification of the specific dynamic evolution of tonic, myoclonic, and no-seizure movement activity [8,26,35].

**Figure 2:**
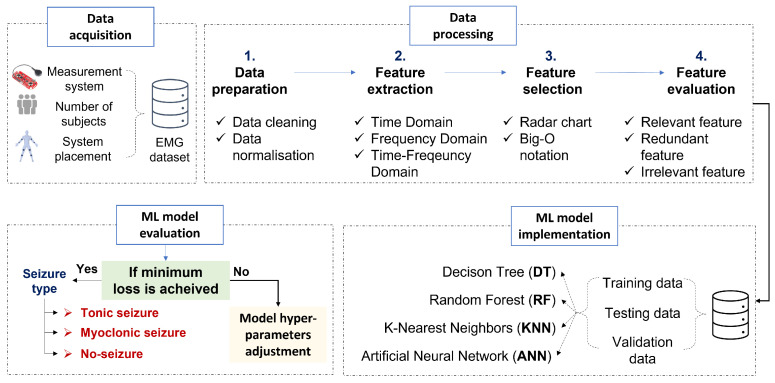
A framework for epileptic movement classification.

For this purpose, a surface EMG dataset is recorded as a first step using a wearable sensor node for tonic, myoclonic, and no-seizure classification. This is followed by a processing step which includes feature extraction and selection methods. Finally, the development of several machine learning algorithms will be described, followed by an evaluation. Figure 2. highlights different blocs used for epilepsy diagnosis, and each bloc will be detailed in the next sections.

## 3. Materials and Methods

### 3.1. System Design

**Figure 3:**
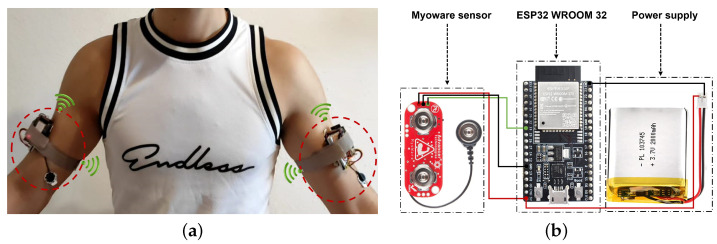
Proposed measurement system. (**a**) Proposed Prototype; (**b**) Prototype specification circuit.

A full control system was developed with high resolution, real-time response, wireless, compact, and high sensitivity insured by WiFi communication with the ESP32 board and a local host (Figure 3a). The components of the proposed prototype are shown in Figure 3b. The system consists of a myoware sensor that converts the surface EMG signal into an easily readable format by measuring, filtering, and rectifying the recorded EMG data. Ag/AgCl electrodes with a 10 mm diameter on self-adhesive supports are used. The recorded sEMG data is transmitted to the ESP32-WROOM-32D microcontroller and then converted to a 12-bit analog-to-digital converter (ADC). A rechargeable Li-ion battery with a capacity of 2400 mAh, 3.7 V, and 8.9 Wh is used as a power supply for all components. The wireless node can continuously transmit raw data for up to 12 h. All components can perfectly fit into the textile hand band with a system length, width, and height equal to 50.5 mm, 38.6 mm, and 33.6 mm, respectively.

### 3.2. sEMG Electrodes Placement

Electrode placement has a noticeable influence on the quality of the measurement, which imposes the necessity to investigate this factor. Commercial Ag/Agcl gel-based electrodes were used to facilitate electrochemical reactions and reduce the skin-electrode interface impedance (less than 10 KΩ) [36]. The considered electrodes permit the charges to pass through the skin-electrode interface without hindrance, which helps the reduction of the signal-to-noise ratio for the recorded biological signals. Furthermore, their low resistivity will help to determine local changes in the impedance of a specific muscle group and prevent overflow of electrical stimulation to other muscle groups [37]. The electrodes were placed in a longitudinal position regarding the muscle fibers to decrease the effect of the subcutaneous fat layer traversed by the current [26].

**Figure 4:**
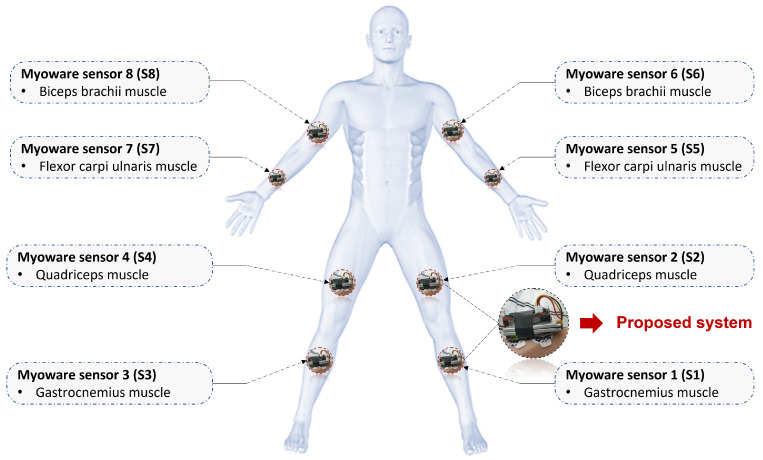
sEMG electrodes placement to classify no-seizure, myoclonic, and tonic seizure movements.

The placement of the proposed wireless sensor node is presented in Figure 4. sEMG electrodes are placed at the recommendation of the Department of child neurology at Hospital Hedi Chaker of Sfax in Tunisia. For that, Ag/Agcl electrodes are placed at a specific position regarding the epileptic seizure movement chosen to be detected. The gastrocnemius flexor carpi ulnaris, biceps brachii, and quadriceps muscles are the selected position for No-seizure, Myoclonic, and Tonic seizure movements distinguish and classification.

### 3.3. sEMG Dataset Description

In order to obtain a sufficient EMG dataset for the classification of the selected epileptic activity motion, 20 healthy subjects simulated tonic, myoclonic, and no-seizure movements. Tonic seizures are characterized by extension of both upper and lower extremities, flexion of upper extremities, and extension of lower extremities. These movements are simulated by having subjects guided by a trainer after watching video recordings of real examples of tonic movements. The videos have been provided by the hospital Hedi Chaker, Sfax, Tunisia. The trainers were asked to correct how they activated the muscles if necessary. Selected subjects belong to the same generation and are aged between 24 and 31, as illustrated in the Table 1.

**Figure 5:**
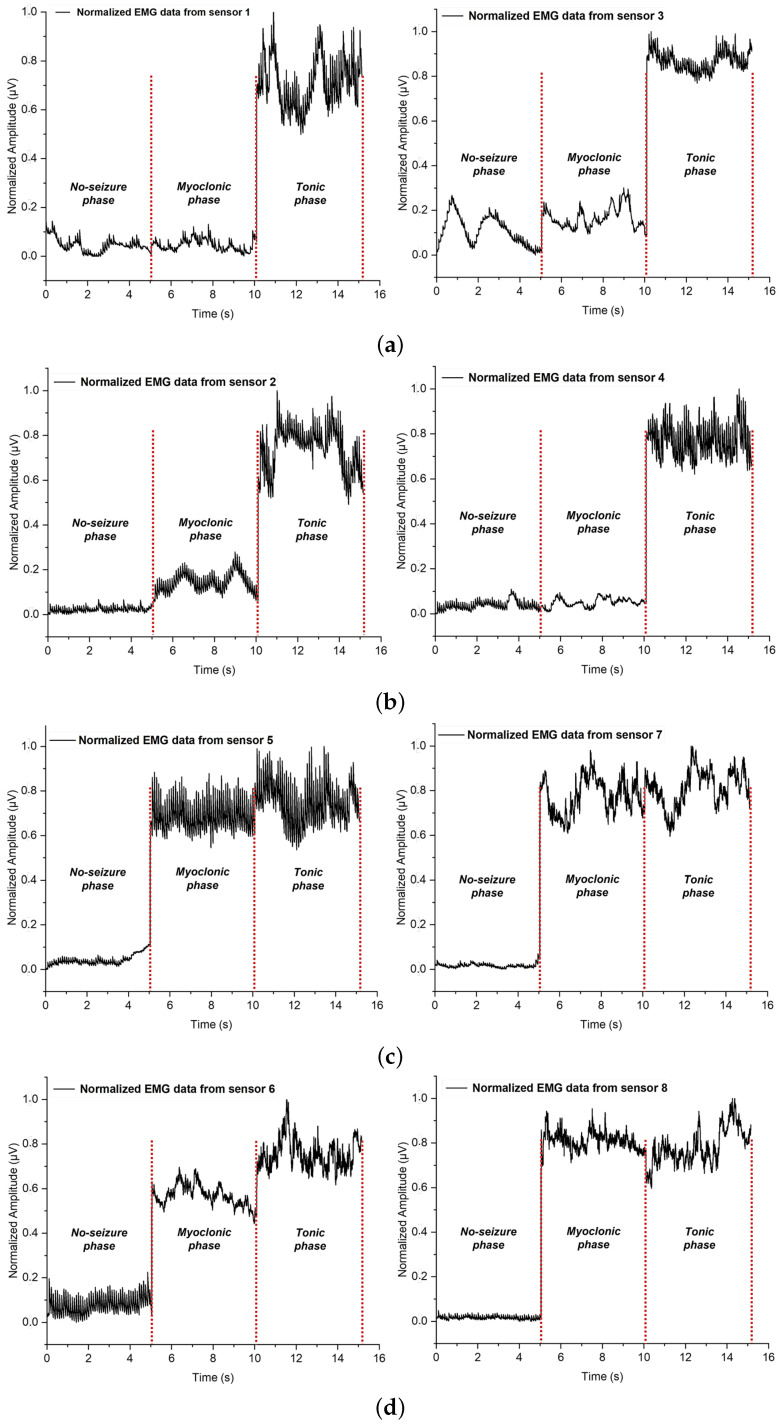
Recorded sEMG signal representation of No-seizure, Myoclonic, and Tonic phases motions. (**a**) Recorded sEMG signal from gastrocnemius muscle; (**b**) Recorded sEMG signal from quadriceps muscle; (**c**) sEMG signal from flexor carpi ulnaris muscle; (**d**) Recorded sEMG signal from biceps brachii muscle.

Figure 5 shows an example of the recorded row sEMG signal for no-seizure, myoclonic, and tonic phase motions. The difference in muscle contraction strength results in a difference in frequency and amplitude range for the three movements. Selected subjects were asked to avoid the direct effect of alcohol and caffeine on muscle contraction by the increase in calcium permeability. They were prohibited from consuming any source of caffeine and alcohol for at least 6 h before the test [37]. They were also asked to fast and stop drinking water for at least 2 h from the beginning of the test and until the end to eliminate the significant change in the bio-impedance quantity caused by food or fluid ingestion [37]. In the same direction, the measurements were performed for each volunteer under the same conditions, e.g., position and measurement duration. After 10 s of maximal contraction in all muscles for the tonic phase, the subjects simulated the myoclonic movement for 2 s with successive epochs of maximal contraction and relaxation in the upper limb muscles. Each subject was asked to simulate ten episodes, with two minutes of rest between trials to avoid muscle charging and ten minutes between motion measurements to avoid muscle fatigue. The four episodes closest to resembling a tonic, myoclonic, or no-seizure motion were chosen for further analysis.

## 4. Data Processing

### 4.1. Feature Extraction

Surface EMG signals have the properties of non-stationary and non-linear signals, making them unusable as raw signals [38,39]. As a result, when these raw signals are used as inputs in sEMG classification, the classifier’s efficiency decreases. In order to improve the performance of the classifier, researchers are using different types of EMG features [40]. Feature extraction transforms short time windows of the raw EMG signal to generate additional information and improve information density [26,27].

During the past decades, numerous different EMG feature extraction methods based on the time domain, frequency domain, and time–frequency domain information have been proposed and explored [8,39]. In general, features in this group are used to detect muscle contraction, muscle action, and onset detection. Sixteen Time Domain Features (TDF) are selected for myoclonic and tonic seizures classification, including Integrated Electromyogram (IEMG), Mean Absolute Value (MAV), Mean Absolute Value 1 (MAV 1), Mean Absolute Value 2 (MAV 2), Sample Square Integral (SSI), Variance (VAR), Temporal Moment (TM), Root Mean Square (RMS), LOG detector (LOG), Waveform Length (WL), Zero Crossing (ZC) [32], Myopulse Percentage Rate (MYOP), Willison Amplitude (WAMP), Kurtosis (KURT), Skewness (SKEW), and Shannon Entropy (SE) [31,32,33,34]. The described features in Table 2, are the most commonly used ones for EMG data processing [27,31,41].

**Figure 6:**
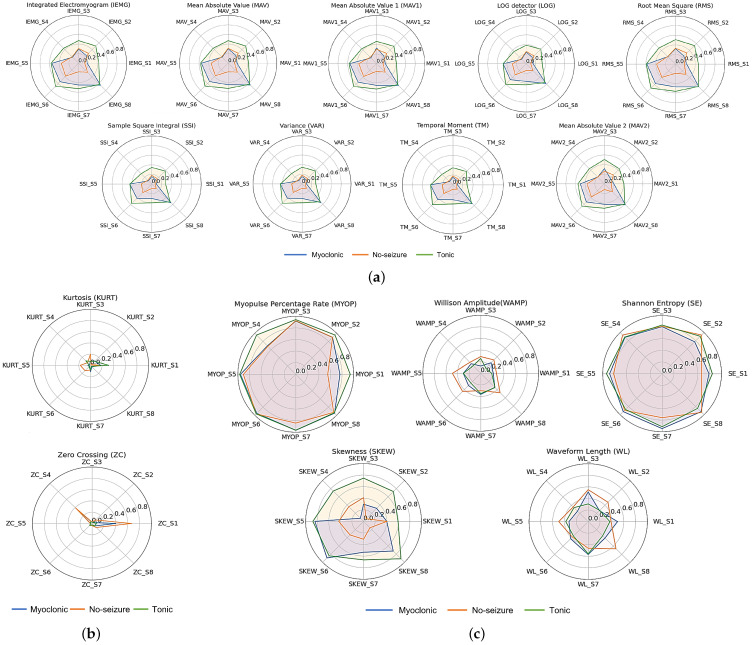
Radar chart of the normalized extracted features. (**a**) Redundant features; (**b**) Irrelevant features; (**c**) Relevant features.

Prior to feature extraction, min-max normalization is performed to compare features initially with variant scales.The radar or spider plot (Figure 6) provide an interesting way to visualize multiple variables in a single graph. This permits to investigate the degree of similarity between multiple classes and their distinguishability. The charts in Figure 6 show the EMG features that provide non-redundant information and build the basis for a principled and interpretable choice of EMG features [30]. To use the output of this topological feature map selection and engineering, we can evaluate measures (such as class separability and robustness) to select from the fundamental and most interesting feature groups the best representative features [30].

The first plot in Figure 6a presents the Integrated EMG feature and the impact of this feature to classify epileptic movements. Each axis presents a sensor (IEMG1, IEMG2, ⋯, IEMG8). From this figure, we can identify which sensor can better contribute to the classification of the movements. For example, for the IEMG plot IEMG_S5 and IEMG_S8 have almost the same value for myoclonic and tonic seizure and therefore, they cannot contribute to differentiate between tonic and myoclonic seizure movements. On the other hand, IEMG_S6 show big differences between myoclonic seizure, tonic seizure, and no-seizure and can contribute well for seizure classification. By interpreting the different extracted features, Figure 6a shows that the radar plots of the IEMG, MAV, MAV1, MAV2, RMS, VAR, TM, LOG, and SSI present redundant information that could be a time-consuming process when training a machine learning or deep learning model because the input to the model depends on the number of extracted features. Also, this mutual information can increase the complexity of a developed classifier. Figure 6b presents the radar plots of the normalized irrelevant features: Kurtosis (KURT) and Zero Crossing (ZC) features. The radar chart of the KURT and the ZC highlights irrelevant information, which means that these features can not distinguish between myoclonic, tonic, and no-seizure movements. As a result, the Kurtosis and Zero Crossing features should be removed. Figure 6c presents the radar plots of the normalized relevant features: WAMP, MYOP, SE, SKEW, and WL. These features can distinguish tonic, myoclonic, and no-seizure activities based on the difference in action potential between the three epileptic movements.

### 4.2. Feature Selection

**Figure 7:**
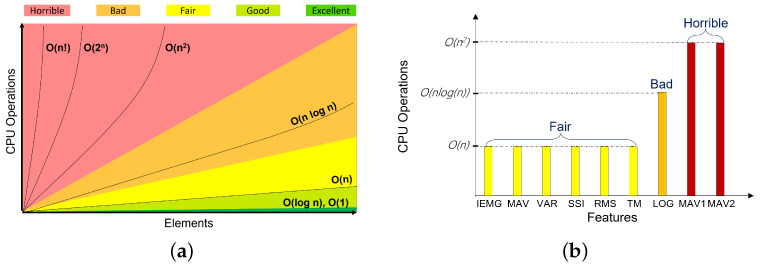
Big-O time complexity chart based feature selection. (**a**) Big-O complexity chart; (**b**) Time complexity.

Feature selection is an essential task in data analysis and information retrieval processing [26]. It reduces the number of features by removing noise and extraneous data [33,42]. Highlighted features in Figure 6a present a similarity in information. Another feature selection method should be performed to choose one feature with low computational time and complexity. This study presents a new method called Big-O Notation, which compares extracted features in terms of time complexity. Time complexity measures how long an algorithm takes to run as a function of the input length. In the same way, space complexity measures how much memory or space an algorithm requires to execute based on input length. Several factors affect space and time complexity, such as the underlying hardware, operating system, CPU, and processor. However, none of these factors are considered when analyzing the algorithm’s performance. Figure 7a presents the Big-O notation chart. It identifies functions according to their growth rates.

**Figure 8:**
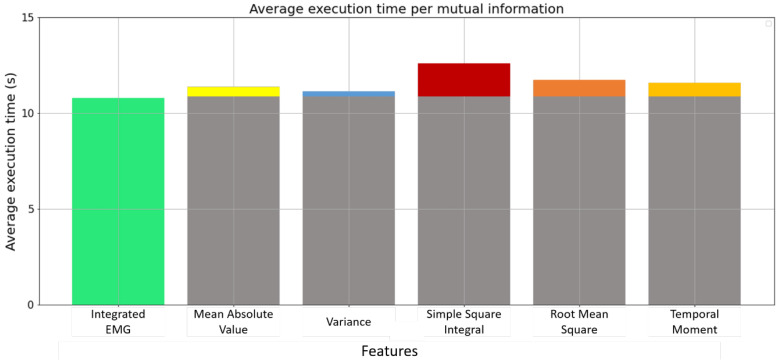
Average execution time per redundant features: IEMG, MAV, VAR, SSI, RMS, and TM.

Different levels of complexity are presented starting from the horrible state (functions can be presented as O(n!), $O(2^{n})$, or $O(n^{2})$), which is the highest time complexity level until the excellent state (functions can be presented as O(logn) or O(1) ) which represents the lowest time complexity level. Based on Figure 7b, LOG, MAV1, and MAV2 present high-level time complexity (bad state for LOG: O(nlogn), Horrible state for MAV1 and MAV2: $O(n^{2})$) compared to other features including the IEMG, MAV, VAR, SSI, RMS, and TM, which presents the same time complexity level O(n). Based on the Big-O feature selection technique, LOG, MAV1, and MAV2 features should be removed. For the six features with the same time complexity level, an average execution time for each feature is used to keep only one feature with a low running time. After ten time trials, Figure 8 shows that the Integrated EMG feature presents the lowest running time compared to other features, with an average execution time of 10.88 s. As a result, the IEMG feature will be used for further processing.

Once the feature set has been evaluated in terms of similarity, insignificant information, execution time, and complexity, six features, including the IEMG, WAMP, MYOP, SE, SKEW, and WL, will be concatenated in the format of vectors and transmitted as inputs to different machine learning classifiers to know the impact of selected features to differentiate between epileptic seizure movements.

## 5. Epileptic Movement Classification Based on Machine Learning Algorithms

Machine learning has proven to be effective in interpreting sEMG signals for different purposes [43], such as to classify gestures [44], to detect muscle fatigue [45], to investigate human–machine interaction [46], and in epilepsy diagnosis or monitoring [47,48]. The possibility of adopting a machine learning approach that learns to interpret the shape of the sEMG signals for assessing muscle-activation onset and offset seems to be a feasible solution [49]. Machine learning is often used as a suitable approach for signal processing. Decision Tree (DT) [50], Random Forest (RF) [51], K-Nearest Neighbors (KNN) [52], and Artificial Neural Network (ANN) [53,54], are the most commonly used classifiers [33,55]. These classifiers are developed and evaluated to classify no-seizure, tonic, and myoclonic epileptic movements based on selected features.

### 5.1. Models Hyperparameter Setup

A hyperparameter is a parameter that measures the learning process using its value. Hyperparameter optimization or tuning is the issue in machine learning to determine a set of ideal hyperparameters for learning models that generalize the model for better accuracy [56]. The performance of the developed machine learning model is dependent on the various hyperparameters such as the criterion, depth of trees for the DT and RF models, the distance and K-neighbor value for the KNN, number of hidden layers, units per layer, epochs, activation function, regularizer, learning rate, batch size, and loss rate for the ANN model. A machine learning engineer can adjust the value of the hyperparameter manually before explicitly training the model. In this study, the used hyperparameters for the DT, RF, KNN, and ANN models are detailed in Table 3. These four algorithms have been implemented to evaluate the classification results according to the investigated dataset and to assess their performance with the change of the implemented dataset. All the results are shown and discussed in the next part.

### 5.2. Machine Learning Models Evaluation

Evaluation of a model is the process of calculating the effectiveness of the data set results. Data manipulation is carried out by the python tool. Table 4 presents various statistics of measurement metrics such as accuracy, precision, recall (sensitivity), and f1-score that are considered to evaluate the performance of all classification algorithms.

**Figure 9:**
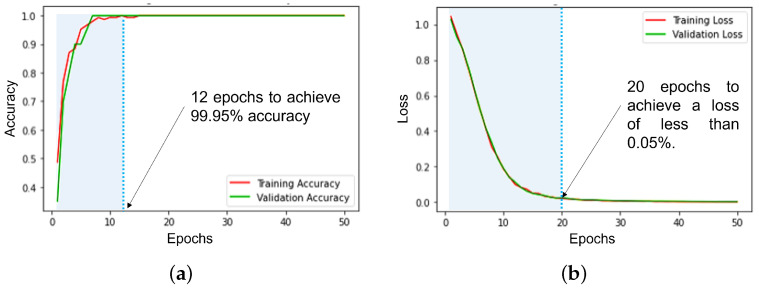
Artificial Neural Network model performance. (**a**) ANN model accuracy over epochs; (**b**) ANN model loss over epochs.

In this study, the dataset was divided into three parts for training, testing, and validation purposes. The dataset is divided into 70% training data, 15% testing data, and 15% validation data. After training the developed models based on the optimal hyperparameters presented in Table 4, the Decision Tree model achieved an average classification accuracy of 91.67% with a precision of 91.90%, recall of 91.67%, and an f1-score of 91.72%. The achieved results by the DT model are approximately the same as for the Random Forest model. The K-Nearest Neighbor model reached an average accuracy of 93.75% with a precision of 94.36%, recall of 93.75%, and an f1-score of 93.66%. However, among the four models assessed, the Artificial Neural Network model performed best. The ANN had an average classification accuracy of 99.95% with a precision of 99.43%, recall of 99.56%, and f1-score of 99.63%. Moreover, Figure 9 shows the training and the validation progress according to the number of epochs in terms of accuracy and loss. The training and validation accuracy reached about 99.95% for the first 10 epochs (Figure 9a). For the loss curve, the training and validation loss went down to about 0.05% with the first 20 epochs (Figure 9b).

The ANN model shows better performance than the KNN model, and the KNN classifier reported better than the RF and the DT models. All achieved results from the developed models are mentioned in Table 5.

To validate the experimental results, sensor importance is added to know the influence of each sensor to classify the selected epileptic movement. The performance of the ANN algorithm in terms of accuracy is evaluated according to the input data. Fifteen different data combinations have been used for the classification, as described in the Table 6. First, a combination of two sensors has been used as inputs for the ANN model. Compared to the accuracy of each sensor combination, the ANN model with 2 sensors placed on both biceps brachii muscles can reach an accuracy of 91.83%. The addition of the number of sensors leads to an increase in the accuracy of the classifier from 94.6% to 96.05% when using a combination of six sensors placed on both gastrocnemius muscles (S1, and S3), quadriceps muscles (S2, and S4), and biceps brachii muscles (S6, and S8). A maximum classification accuracy of 99.95% is achieved while using the combination of the eight proposed sensors. The results in Table 6 show, that the ANN model with the eight sensors is necessary for accurate epileptic movements classification. Reducing the number of sensors leads also to a reduction in classification accuracy.

Even though multiple sensors may pose challenges, they are very important to realize the necessary accuracy enabling the effective detection and classification of motor seizures. At the same time, it is also important to consider the practicability and usability of the system in real-life scenarios and explore ways to make the sensors less stigmatizing and more comfortable for patients by developing smaller and more discreet sensors or finding ways to integrate the sensors into existing clothes or accessories.

## 6. Conclusions

The study in this paper shows, that the analysis of muscle activity can provide valuable information for seizure classification. In a novel approach, we propose to track epileptic seizures with eight surface electromyography signals (sEMG) measured at dedicated placements on human limbs. We propose to use a machine learning model to analyze and classify two motor seizures for epileptic subjects. Measurements on 20 subjects imitating tonic, myoclonic, and no-seizure movements support this study. Features of the EMG signals, such as maximum class separability, robustness, and computational complexity, lead to very good classification performance. The conclusion was that the IEMG, MYOP, WAMP, SE, SKEW, and WL feature highly separable epileptic movements. The ANN model achieved the greatest classification accuracy rate of 99.95% in comparison to classification algorithms based on decision tree, random forest, k-nearest neighbors, and artificial neural networks.

This work proves that surface electromyography is promising for the classification of myoclonic and tonic epileptic seizures. The investigation is mainly based on measurements during movements imitating the movements observed during seizures. Medical doctors report, that the muscle contractions during seizure attacks are expected to be much stronger, so that the classification for real non-healthy subject becomes even easier. This study serves as a technical feasibility investigation, paving the way for clinical trials. In future further studies need to be conducted to expand the dataset with further epileptic seizure movements (absence seizure). Clinical studies need to be conducted to record data from the pediatric patients and to explore longer monitoring periods to capture also infrequent epileptic seizure movements.

## Figures and Tables

**Table 1:** Selected subjects specification.

Subject	Gender	Age	Weight (kg)	High (m)	Subject	Gender	Age	Weight (kg)	High (m)
1	male	23	63	1.83	11	female	26	66	1.68
2	male	24	70	1.85	12	female	24	92	1.86
3	male	27	63	1.75	13	female	24	62	1.64
4	male	25	84	1.78	14	female	25	65	1.70
5	male	25	81	1.77	15	female	25	61	1.64
6	male	25	74	1.83	16	female	24	58	1.72
7	male	25	82	1.78	17	female	27	75	1.71
8	male	24	97	1.80	18	female	27	62	1.68
9	male	27	63	1.83	19	female	24	53	1.62
10	male	26	89	1.82	20	female	26	58	1.76

**Table 2:** Extracted Time Domain Features (TDF) from EMG signal.

Abbreviation	Feature	Equation
IEMG	Integrated EMG	$IEMG=\sum_{k=1}^{N}\left|S_{k}\right|$, Here N denotes the length of the signal and $S_{k}$ represents the sEMG signal in a segment.
MAV	Mean Absolute Value	$MAV=\frac{1}{N}\sum_{k=1}^{N}\left|S_{k}\right|$
MAV 1	Mean Absolute Value 1	$MAV1=\frac{1}{N}\sum_{k=1}^{N}\omega_{n}\left|S_{k}\right|$, $\omega_{k}=\left\{\begin{array}{c} 1,\;0.25N\leq k\leq 0.75N\\ 0.5,\;otherwise \end{array}\right.$
MAV 2	Mean Absolute Value 2	$MAV2=\frac{1}{N}\sum_{k=1}^{N}\omega_{k}\left|S_{k}\right|$, $\omega_{k}=\left\{\begin{array}{c} 1,\;0.25N\leq k\leq 0.75N\\ \frac{4k}{N},\;0.25N>k\\ \frac{4(k-N)}{N},\;0.75N<k \end{array}\right.$
SSI	Simple Square Integral	$SSI=\sum_{k=1}^{N}{S_{k}}^{2}$
VAR	Variance	$VAR=\frac{1}{N-1}\sum_{k=1}^{N}{S_{k}}^{2}$
TM	Temporal Moment	$TM=|\frac{1}{N}\sum_{k=1}^{N}{S_{k}}^{3}|$
RMS	Root Mean Square	$RMS=\sqrt{\frac{1}{N}\sum_{k=1}^{N}{S_{k}}^{2}}$
LOG	LOG detector	$LOG=e^{1/N\sum_{k=1}^{N}log\left|S_{k}\right|}$
WL	Waveform Length	$WL=\sum_{k=1}^{N-1}\left|S_{k+1}-S_{k}\right|$
ZC	Zero Crossing	$ZC=\sum_{k=1}^{N-1}\left[sgn\left(S_{k}\cdot S_{k+1}\right)⋂\left|S_{k}-S_{k+1}\right|\geq 0\right]$, $sgn(S)=\left\{\begin{array}{c} 1,\;S\geq threshold\\ 0,\;otherwise \end{array}\right.$
MYOP	Myopulse Percentage Rate	$MYOP=\frac{1}{N}\sum_{k=1}^{N}f\left(\left|S_{k}\right|\right)\geq threshold$, $f\left(S\right)=\left\{\begin{array}{c} 1,\;S\geq threshold\\ 0,\;otherwise \end{array}\right.$
WAMP	Willison Amplitude	$WAMP=\sum_{k=1}^{N-1}f\left(\left|S_{k+1}-S_{k}\right|\right)>threshold$, $f\left(S\right)=\left\{\begin{array}{c} 1,\;S\geq threshold\\ 0,\;otherwise \end{array}\right.$
KURT	Kurtosis	$KURT=\frac{1}{N}\frac{\sum_{k=1}^{N}{\left(S_{k}-\mu \right)}^{4}}{\sigma^{4}}$
SKEW	Skewness	$SKEW=\frac{1}{N}\frac{\sum_{k=1}^{N}{\left(S_{k}-\mu \right)}^{3}}{\sigma^{3}}$
SE	Shannon Entropy	$SE=-\sum_{k=1}^{N}S_{k}log\left(S_{k}\right)$

**Table 3:** Selected hyper-parameters for classification models.

Predictive Model	Hyperparameter	Tuned to
DT	Criterion	Gini, Entropy
	Depth of trees	4
RF	Criterion	Gini, Entropy
	Decision trees	2
	Maximum features	Auto
KNN	K-neighbour	K = 3
	Distance	Euclidean
ANN	Batch size	20
	Epochs	50
	Hidden layers	1
	Neurons	64
	Activation function	Softmax
	Learning rate	$10^{-5}$
	Optimizer	Adam
	Loss rate	Categorical Crossentropy
	Regularizer	L2 regularizer

**Table 4:** Performance metrics for classifiers evaluation.

Metric	Description
Accuracy	Measure of the model’s correct predictions.
Precision	Determine the classifier’s ability to deliver accurate positive predictions.
Recall	Probability of a positive test, conditioned on truly being positive.
F1-score	Weighted average of precision and recall.

**Table 5:** Machine learning models evaluation.

Predictive Model	Accuracy (%)	Precision (%)	Recall (%)	F1-Score (%)
DT	91.67	91.90	91.67	91.72
RF	91.67	92.13	91.67	91.65
KNN	93.75	94.36	93.75	93.66
ANN	99.95	99.43	99.56	99.63

**Table 6:** Classification results over different sensor combination.

Sensors Combination	S1	S2	S3	S4	S5	S6	S7	S8	Accuracy (%)
2					x		x		85.51
	x		x						87.75
		x		x					88.50
						x		x	91.83
4	x	x	x	x					90.00
	x		x			x		x	92.40
		x		x	x		x		93.64
	x		x		x		x		93.82
					x	x	x	x	94.27
		x		x		x		x	94.60
6	x		x		x	x	x	x	93.84
	x	x	x	x	x		x		94.21
		x		x	x	x	x	x	95.59
	x	x	x	x		x		x	96.05
8	x	x	x	x	x	x	x	x	99.95

## Data Availability

Not applicable.
